# Risk factors and predicted distribution of visceral leishmaniasis in the Xinjiang Uygur Autonomous Region, China, 2005–2015

**DOI:** 10.1186/s13071-019-3778-z

**Published:** 2019-11-08

**Authors:** Fangyu Ding, Qian Wang, Jingying Fu, Shuai Chen, Mengmeng Hao, Tian Ma, Canjun Zheng, Dong Jiang

**Affiliations:** 1 State Key Laboratory of Resources and Environmental Information System, Institute of Geographical Sciences and Natural Resources Research, Chinese Academy of Sciences, Beijing, 100101 China; 2 College of Resources and Environment, University of Chinese Academy of Sciences, Beijing, 100049 China; 3 National Institute for Viral Disease Control and Prevention, Chinese Center for Disease Control and Prevention (China CDC), Beijing, 102206 China; 4 Key Laboratory of Carrying Capacity Assessment for Resource and Environment, Ministry of Land & Resources, Beijing, 100101 China

**Keywords:** Visceral leishmaniasis, Spatiotemporal patterns, Environmental niche, Infection risk

## Abstract

**Background:**

Visceral leishmaniasis (VL) is a neglected disease that is spread to humans by the bites of infected female phlebotomine sand flies. Although this vector-borne disease has been eliminated in most parts of China, it still poses a significant public health burden in the Xinjiang Uygur Autonomous Region. Understanding of the spatial epidemiology of the disease remains vague in the local community. In the present study, we investigated the spatiotemporal distribution of VL in the region in order to assess the potential threat of the disease.

**Methods:**

Based on comprehensive infection records, the spatiotemporal patterns of new cases of VL in the region between 2005 and 2015 were analysed. By combining maps of environmental and socioeconomic correlates, the boosted regression tree (BRT) model was adopted to identify the environmental niche of VL.

**Results:**

The fitted BRT models were used to map potential infection risk zones of VL in the Xinjiang Uygur Autonomous Region, revealing that the predicted high infection risk zones were mainly concentrated in central and northern Kashgar Prefecture, south of Atushi City bordering Kashgar Prefecture and regions of the northern Bayingolin Mongol Autonomous Prefecture. The final result revealed that approximately 16.64 million people inhabited the predicted potential infection risk areas in the region.

**Conclusions:**

Our results provide a better understanding of the potential endemic foci of VL in the Xinjiang Uygur Autonomous Region with a 1 km spatial resolution, thereby enhancing our capacity to target the potential risk areas, to develop disease control strategies and to allocate medical supplies.

## Background

Visceral leishmaniasis (VL), also known as kala-azar, is a vector-borne disease that has a broad distribution throughout many temperate, subtropical and tropical areas of the world [1, 2]. The disease is most prevalent in the Mediterranean basin, Brazil, the northern part of the Indian subcontinent and the northeastern countries of Africa and is associated with approximately 0.5 million new cases and 3.3 million disability-adjusted life years, resulting in an estimated mortality of 200,000–400,000 people per year worldwide [3, 4]. VL is caused by the trypanosomatid protozoan parasite *Leishmania*, which is spread to humans by the bites of infected female phlebotomine sand flies [5, 6]. When the disease occurs during pregnancy and without appropriate treatment, it may lead to high-grade anaemia, spontaneous loss and congenital leishmaniasis because of transplacental transfer of parasites [7]. In terms of mortality and morbidity, the fatal parasitic disease was ranked ninth in a global analysis of infectious diseases by the World Health Organization [3].

In China, people have been struggling against VL for at least 120 years, dating back to the late period of the Qing Dynasty [8]. In 1904, the first case of VL was formally reported, and additional cases were reported during the next decade [8, 9]. Then, the endemic disease, along with other infectious and parasitic diseases, was rampant in the vast rural areas north of the Yangtze River, mainly distributed in Anhui, Jiangsu, Henan, Shaanxi, Gansu, Shandong, Hebei and Liaoning provinces during the subsequent period from 1920 to 1940 [10, 11]. In the 1940s, the problem became more serious due to the continuation of the Second World War and the lack of preventive measures [1, 12]. In 1951, a detailed survey conducted by the government showed that VL had spread far more widely than before in more than 660 counties/cities of 16 provinces; it was associated with at least 530,000 cases, and the incidence rate in each county ranged from 10/100,000 to 500/100,000 [12, 13]. At that time, a national comprehensive control programme was designed and implemented stringently by the government of the People’s Republic of China at all administrative levels to eliminate VL from most areas of endemicity, resulting in a steady decline in the number of reported cases during the subsequent decades [10, 14–16]. Since the late 1980s, national programmes for developing western and northwestern China were implemented, which provided suitable habitats for the transmission of VL and caused a resurgence of the disease in these regions [17]. For instance, there were 2629 new cases officially reported in the 1990s, and approximately 38.8% of them occurred in the Xinjiang Uygur Autonomous Region.

**Fig. 1:**
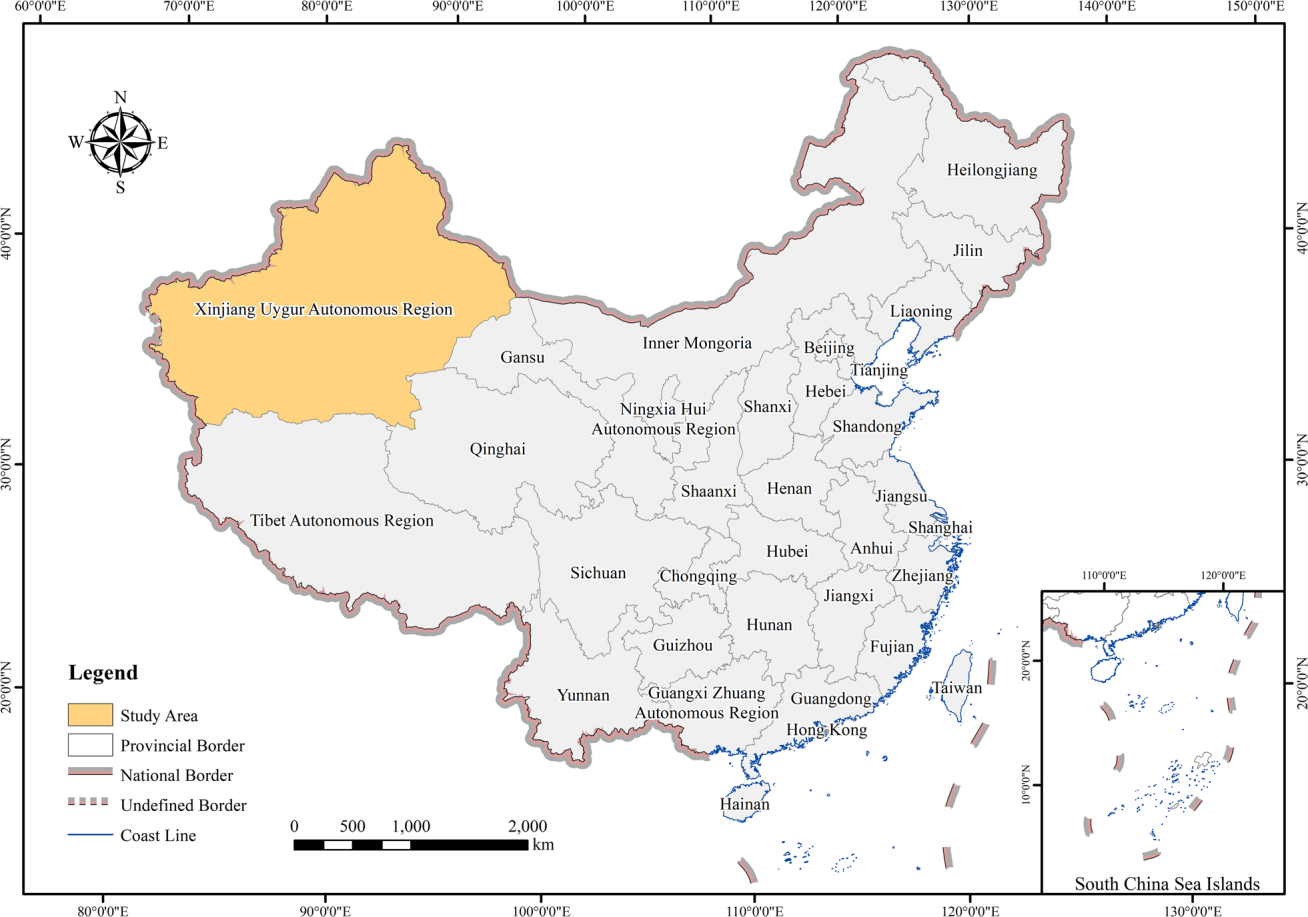
The location of the Xinjiang Uygur Autonomous Region in China. The administrative boundary dataset was downloaded freely from Resource and Environment Data Cloud Platform (REDCP) (http://www.resdc.cn). The figure was generated specifically for this research using ArcGIS10.2

The Xinjiang Uygur Autonomous Region is one of the VL endemic foci in China due to the unique geographical and ecological environment [18]. Since 2000, nearly 100 new cases have been reported each year in the region, with an increasing trend year by year [1], which revealed that the health burden of VL was underestimated (Fig. 1). Therefore, the objectives of this study were to analyse the spatiotemporal dynamic patterns of VL cases in Xinjiang from 2005 to 2015, to identify the environmental niche of VL, to map the potential zones of VL infection risk at high spatial resolution (1 km) and to provide novel insights into the health burden imposed by VL in the Xinjiang Uygur Autonomous Region.

## Methods

A boosted regression tree (BRT) modelling technology that has been useful for analysing other vector-borne diseases such as dengue [19] and yellow fever [20] was adopted to produce maps of potential VL infection risk in the Xinjiang Uygur Autonomous Region. Compared with other machine learning models (i.e. support vector machines and backward propagation neural networks), the BRT model has a better explanatory power and ability to handle complex non-linear relationships with given environmental and socioeconomic covariates [21, 22]. This modelling technology required three key information elements: (i) a suite of gridded layers on environmental and socioeconomic correlates of VL; (ii) a comprehensive dataset of VL occurrence records with detailed address information; and (iii) pseudo-absence records. A detailed description of the BRT model can be found elsewhere [23, 24]. In the present study, all data were transformed into the same geographical coordinate system (WGS-84) and the same projected coordinate system (Albers Conical Equal Area) and unified to a raster with a 1 × 1 km spatial resolution. In the process of data preprocessing and output, Python 2.7.0 (https://www.python.org/) combined with the Geospatial Data Abstraction Library (GDAL) 2.1.0 (http://www.gdal.org/) and Proj4 5.0.0 (https://proj4.org/) were employed.

### Environmental and socioeconomic correlates

In China, VL is known to have anthroponotic and zoonotic transmission cycles, which differ in their transmission characteristics: the former is transmitted from humans to vectors to humans, and the latter is transmitted from animals to vectors to humans [2]. For both zoonotic VL and anthroponotic VL, phlebotomine sand flies as the vector play an important role in the spread of the disease. The distribution of the vector is determined by various key environmental and socioeconomic correlates; thus, the spatiotemporal patterns of VL are considered in relation to these covariates. In the present study, several related covariates were adopted to map the potential transmission risk zones of VL in the Xinjiang Uygur Autonomous Region. Detailed information on the related covariates is listed in Table 1.

**Table 1:** Environmental and socioeconomic correlates

Factor	Parameter	Data source
Ecological	Normalized difference vegetation index (NDVI)	Global Inventory Modelling and Mapping Studies (GIMMS) group
	Land cover	European Space Agency (ESA)
Climatic	Annual cumulative precipitation (mm)	China Meteorological Data Service Center (CMDC)
	Mean temperature (°C)	
	Relative humidity (%)	
Terrain	Elevation (m)	Shuttle Radar Topography Mission (SRTM)
Socioeconomic	Urban accessibility (hour)	European Commission Joint Research Center (ECJRC)
	Night-time light	Earth Observation Group, National Oceanic and Atmospheric Administration (NOAA)

#### Ecological factors

Vegetation plays an important role in sand fly habitat and survival by providing the necessary sugar resource and maintaining the necessary moisture profile for both immature and adult sand flies [25, 26]. Vegetation canopy cover could reduce evaporation, decrease sub-canopy wind speed and protect certain areas from direct sunlight, providing a comfortable habitat for the survival of the dipterans [19]. In addition, vegetation is an important food for many mammals, serving as a platform for sand flies to feed on passing mammals [27]. In the present study, we adopted the NDVI as a potential indicator of vegetation canopy cover at a given location. From the GIMMS group (https://ecocast.arc.nasa.gov/), the advanced very high-resolution radiometer (AVHRR) NDVI dataset spanning from 2005 to 2015 was obtained. Based on the AVHRR NDVI dataset, we used the maximum value composition technique and the mean method to extract information about the average value for each gridded cell.

Previous studies have also illustrated that there is a link between VL and land cover [3]. For instance, the infection rate of VL is often highest among people living at the edge of natural foci, i.e. forests and deserts. The land cover map from January to December 2009 with 0.3 × 0.3 km spatial resolution was downloaded from the website (http://due.esrin.esa.int/) of the Data User Element of ESA, which was processed by ESA and the University of Louvain and made available to the public. In this study, land cover was adopted as a key explanatory variable in the distribution of VL cases.

#### Climatic factors

Several studies have revealed that temperature, precipitation and humidity have strong effects on the ecology of vectors and reservoir hosts by influencing their survival, population sizes and distribution [26, 27]. Temperature has often been identified as an important factor influencing sand fly metabolism, developmental times and fecundity [28, 29]. For example, all female *Phlebotomus papatasi* die before laying eggs at 15 °C, while the lifespan of the adult increases with decreasing temperature within a range of 18–32 °C [30]. Moreover, studies have shown that temperature could also influence the development of several species of *Leishmania* in the natural vectors [29]. Precipitation and humidity have been shown to play a prominent role in shaping the distribution of VL by influencing the breeding and resting of the vector [31]. For instance, ecotopes occupied by immature phlebotomines are usually organically rich, moist areas (i.e. the rainforest floor).

From the website of the CMDC (http://data.cma.cn), the dataset (V3.0) of daily values of climate data from Chinese surface stations was downloaded. Based on the point-level meteorological dataset, ANUSPLIN-SPLINA software was employed to produce a series of meteorological raster layers. Then average values of three meteorological factors were calculated for each gridded cell during the period from 2005 to 2015, including mean annual temperature, mean annual relative humidity and annual cumulative precipitation.

#### Terrain factor

Previous studies have illustrated that there is a link between terrain and several vector-borne diseases [20, 32]. A controlled trial conducted by Hlavacova et al. [29] suggested that *Leishmania infantum* and *L. braziliensis* could spread to higher altitudes than *L. peruviana* could. Although the relationship has not been understood, we assumed that topography may restrict the vector to certain geographical areas. In this study, an elevation dataset generated by the SRTM was used as a good measure for topography, which was downloaded from the website of the CGIAR Consortium for Spatial Information (http://srtm.csi.cgiar.org) [33].

#### Socioeconomic factors

There is a strong but complex association between VL and socioeconomic covariates [2, 34, 35]. On the one hand, a local study conducted by Boelaert et al. [36] illustrated that low-income populations are most vulnerable to VL, as poor housing conditions and unhealthy habitats increase sand fly breeding and resting sites. On the other hand, poverty is linked with poor nutrition, which compromises the immunity of poor populations and increases the risk that VL infection will progress to the clinically manifested disease [3, 37]. In the present study, night-time light satellite imagery with a 1 km spatial resolution was adopted to represent the geographic variation of poverty due to a good positive linear correlation between the two [38]. The stable light layers of night-time light satellite imagery spanning from 2005 to 2013 were downloaded from the NOAA Earth Observation Group (https://ngdc.noaa.gov/). Based on the 9 years of the night-time light dataset, the mean across all years for each gridded cell in the Xinjiang Uygur Autonomous Region was computed.

VL are often associated with population movements. For example, the introduction of nonimmune people into areas with existing endemic foci may result in new infection cases [3]. Several studies on other vector-borne diseases (i.e. scrub typhus, zika and dengue) also revealed that human movement aided disease transmission through a series of cascading effects, particularity in highly accessible regions towards which people tend to gravitate [19, 21, 39]. In this study, an urban accessibility dataset estimating the travel time to the nearest city with a population of 50,000 people or more was adopted as an approximate measure index to account for patterns of human movement. The approximately 1 × 1 km gridded dataset was obtained from the website of the ECJRC (http://forobs.jrc.ec.europa.eu/).

### Occurrence and pseudo-absence records

The known comprehensive human infection cases of VL in the Xinjiang Uygur Autonomous Region spanning 2005–2017 were obtained from the Chinese Center for Disease Control and Prevention (CDC) (http://www.chinacdc.cn/). It should be noted that clinically diagnosed and laboratory-confirmed human infection cases reported during 2005–2015 were adopted in the modelling process, and suspected cases of VL were not used in the present study due to their own uncertainty. The geoposition information on these cases is at least accurate at the township level, and most can be detailed at the village level. By combining Google Earth (http://earth.google.com/) with the geopositioning information of the cases, VL occurrences were manually geopositioned to the point level with coordinates and checked to ensure that the coordinates were plausible. Then, these point-level occurrence records were rasterized to grid cells with a 1 km spatial resolution to match the spatial resolution of related environmental and socioeconomic covariates. In total, 603 grid units derived from the point-level occurrence records were obtained, which were labelled as high-risk samples, representing related environmental and socioeconomic conditions suitable for the transmission of VL.

BRT modelling technology requires both occurrence and pseudo-absence records to identify the realized niche of diseases. The latter have previously been shown to have a great effect on model accuracy [19, 40], but there is no general consensus on how to generate pseudo-absence records. Compared with occurrence records, pseudo-absence records were used to provide a sample set of conditions in places where VL cases were not observed during the period from 2005 to 2015. In this study, 603 grid units where VL was not present were randomly selected as pseudo-absence records from the counties where cases of VL infection were reported during the period from 2005 to 2015, which were labelled as low-risk samples.

### Modelling approach

Version 3.3.3 of the 64-bit version of R was employed to build the model and assess the prediction performance. In the R statistical programming environment, the extension packages included *dismo* and *gbm* packages [41, 42]. Based on occurrence and pseudo-absence records, the BRT modelling procedure was used to fit VL along with a range of environmental and socioeconomic variables. To improve the performance of BRT modelling technology, we repeated the process of randomly selecting pseudo-absence data 300 times. During each random process of selecting pseudo-absence data, we divided all risk samples into training and validation samples, and the former and latter accounted for 75% (*n* = 905) and 25% (*n* = 301) of the total samples (*n* = 1206), respectively. According to the suggestion of Messina et al. [43], the main tuning parameters were set (tree.complexity = 4; learning.rate = 0.005; bag.fraction = 0.75; step.size = 10; cv.folds = 10; max.trees = 10000), and the other tuning parameters of the algorithm were held at their default values. In the process of training the model, a ten-fold cross-validation method was applied to prevent over-fitting. An ensemble of 300 BRT models was fitted, and we performed analyses for the predictive performance of BRT models using the area under curve (AUC) statistic. Relative contribution (RC) indicator was used to reflect the contribution of each predictor.

## Results

### Spatiotemporal patterns

**Fig. 2:**
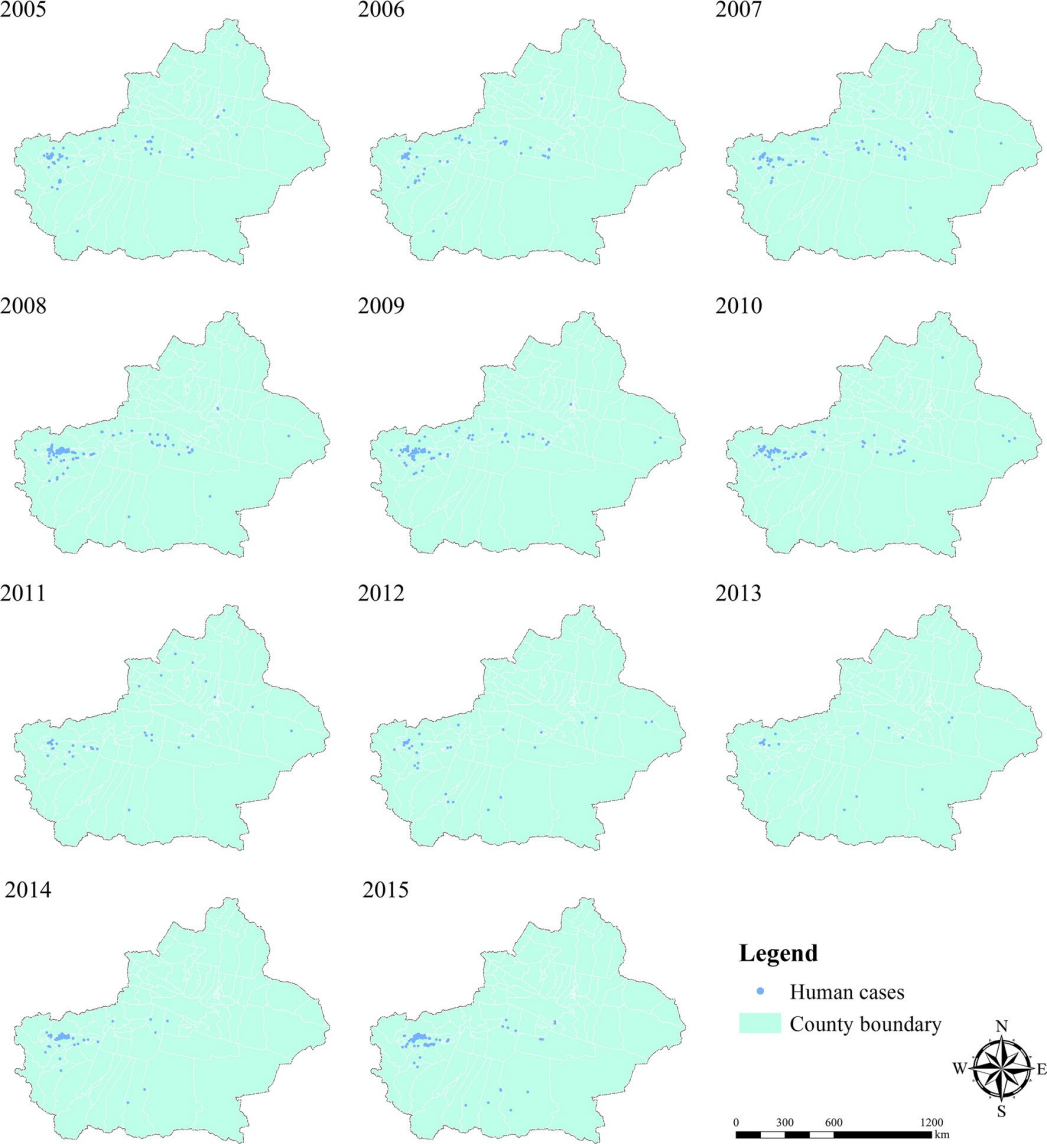
The geographical distribution of VL cases in the Xinjiang Uygur Autonomous Region from 2005 to 2015. The VL infection cases were obtained from CDC, and all the data analyzed in this study were de-identified to protect patient confidentiality. The provincial-level and county-level administrative boundary dataset were downloaded freely from REDCP. The figure was generated specifically for this research using ArcGIS10.2

Figure 2 depicts the spatiotemporal patterns of clinical diagnoses and laboratory-confirmed cases of VL infection during the period from 2005 to 2015. According to the statistics, the number of VL infection cases occurring in 2005 was 154, mainly concentrated in the Kashgar Prefecture and Aksu Prefecture. In 2006, the number of new cases officially reported decreased slightly, with 128 cases. There was a significant increase in the number of new cases in the Xinjiang Uygur Autonomous Region, increasing from 131 in 2007 to 340 in 2008. During the period from 2009 to 2013, the number of new VL infection cases showed a gradual decline, with 296, 150, 61, 43 and 23 cases, sequentially. In the next two years, the number of new VL cases rebounded rapidly and reached a new high value of 397 in 2015. Overall, the new VL infection cases reported from 2005 to 2015 have obvious spatial clustering, and most cases are concentrated in Kashgar Prefecture, Aksu Prefecture and Bayingolin Mongol Autonomous Prefecture (Fig. 2). In addition, sporadic VL cases occurred occasionally in the rest of the regions of the Xinjiang Uygur Autonomous Region.

### Environmental niche

**Fig. 3:**
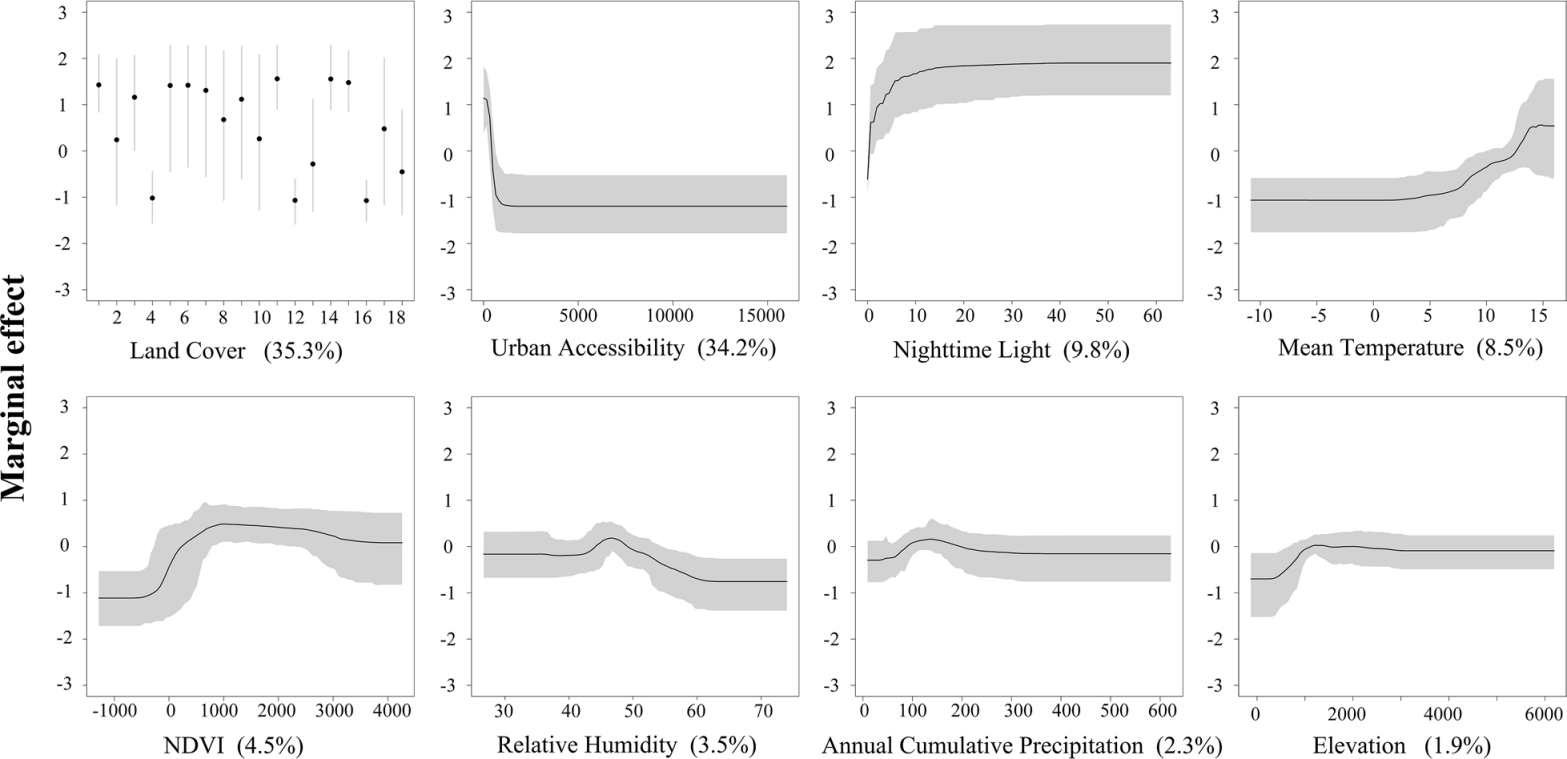
Marginal effect curves of each predictor over all 300 BRT ensembles

Figure 3 identifies the environmental niche of VL in the Xinjiang Uygur Autonomous Region, which is derived from all 300 BRT ensembles. The black lines represent the mean effect curves over all 300 BRT ensembles, and the shaded areas envelope the mined relationships to each predictor from all BRT ensembles within the 95% confidence interval (Fig. 3). Examination of the marginal effect curves reveals that land cover is the most important predictor contributing to the occurrence map, accounting for 35.30% (SE: 12.32%) of variation explained by the ensemble BRT models. The names of the land cover types are described in Additional file 1: Table S1. The socioeconomic correlates, namely, urban accessibility and nighttime light, are also the important predictors contributing to the ensemble BRT models, accounting for 34.16% (SE: 12.26%) and 9.83% (SE: 3.33%), respectively. The other main predictors are mean temperature (RC: 8.51%, SE: 2.01%, positive association), NDVI (RC: 4.47%, SE: 1.68%, positive association), relative humidity (RC: 3.46%, SE: 1.41%, complex association), annual cumulative precipitation (RC: 2.33%, SE: 1.02%, complex association) and elevation (RC: 1.94%, SE: 0.94%, positive association).

### Potential infection risk zones

**Fig. 4:**
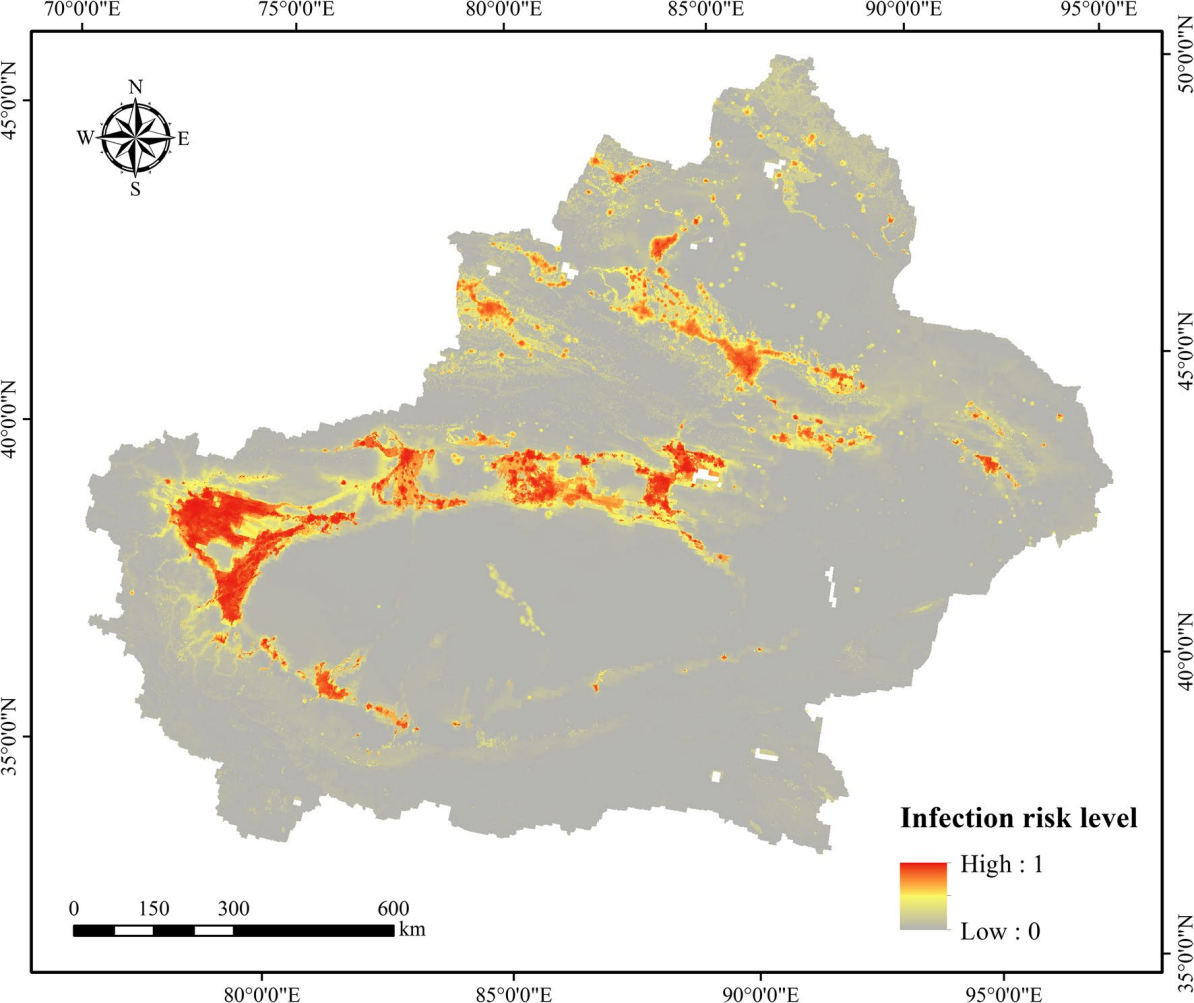
The geographical distribution of the predicted potential VL infection risk zones, with the risk level ranging from 0 (grey) to 1 (red). The figure was generated by calculating the mean prediction across all 300 BRT ensembles for each gridded cell, which was produced specifically for this research using ArcGIS10.2

According to the explored environmental niches among predictors and VL occurrence, the fitted ensemble BRT models were employed to estimate potential infection risk zones in the Xinjiang Uygur Autonomous Region. The map shows that the predicted highest infection risk areas are mainly concentrated in several regions, including central and northern Kashgar Prefecture, south of Atushi City bordering Kashgar Prefecture and regions of the northern Bayingolin Mongol Autonomous Prefecture (Fig. 4). The boundaries of the main administrative districts are shown in Additional file 1: Figure S1. The predicted risk for VL infection is also high in some regions of Hotan Prefecture, extending from northwest regions (central Pishan County, southern Moyu County and northern Hotan County) to central regions (southern Lop County, central Qira County and central Yutian County). In Aksu Prefecture, the zones predicted to have higher infection risk present two spatial clusters, located in the midwestern and eastern parts of the region. The predicted risk level is also high in Urumqi city and its surrounding areas (Changji Hui Autonomous Prefecture and Turpan Prefecture). For the northern part of Xinjiang, the distribution of the predicted infection risk areas is relatively fragmented. Overall, the fitted ensemble BRT models obtained high predictive accuracy within the Xinjiang Uygur Autonomous Region (training data 10-fold cross-validation AUC: 0.976, SE: 0.006; validation data AUC: 0.976, SE: 0.005).

## Discussion

By combining maps of environmental and socioeconomic correlates with comprehensive infection records, this study estimated potential infection risk zones of VL at 1 × 1 km spatial resolution grids in the Xinjiang Uygur Autonomous Region. The final predicted map revealed that the potential high infection risk zones were mainly concentrated in central and northern Kashgar Prefecture, south of Atushi City bordering Kashgar Prefecture and regions of the northern Bayingolin Mongol Autonomous Prefecture. Based on the standard deviation values calculated for each grid across the model ensemble, we also quantified the model uncertainty in spatial predictions of VL infection risk, as shown in Additional file 1: Figure S2. The uncertainty map illustrates that there is low prediction uncertainty in the Xinjiang Uygur Autonomous Region.

To convert the continuous VL infection risk map into a binary surface (i.e. high or low risk), the threshold value of 0.5 was used in the present research. Based on the Gridded Population of the World Version 4 population density for the year 2015 [44], we also estimated that approximately 16.64 million people inhabited the predicted potential infection risk areas in the Xinjiang Uygur Autonomous Region. Additional file 1: Table S2 illustrates the top six prefecture-level administrative units contributing to these populations in the predicted high-risk zones. For example, Kashgar Prefecture has the most people living in areas that are suitable for VL transmission at 3.95 million people, followed by Urumqi city (3.69 million people), Ili Kazakh Autonomous Prefecture (1.94 million people) and Aksu Prefecture (1.73 million people), which provides an important reference for further calculations of the public health burden imposed by VL. It is also important to recognize that the probability that people are infected with VL is different even in the most receptive environments due to differences between individuals, such as living habits and immunity [45, 46]. In the predicted high-risk zones, it is necessary to encourage people to use insecticide-treated bed nets to avoid contact with phlebotomine sand flies.

There are several published studies on risk mapping for VL. Pigott et al. [27] combined evidence consensus maps with a statistical modelling framework to generate the first distribution map of VL on a global scale. Rajabi et al. [47, 48] employed several spatial modelling techniques to map the potential risk areas of VL in the countries of southern Caucasus. Iliopoulou et al. [49] used a spatial regression model to produce a risk map for VL in the Attica region, Greece. The purpose of the above studies was to generate a risk map for VL in the study area based on explanatory variables and modelling techniques. The first three studies showed relative risk levels by values between 0–1, while the last study used predictive number of human cases as a measure of risk for VL. Compared with the modelling techniques adopted in these studies, the BRT modelling framework adopted in this study could explore the complex relationships between VL and related covariates and avoid over-fitting. For instance, the probability of occurrence is positively correlated with mean temperature and NDVI. However, it should be noted that this study has some limitations. Although some factors (i.e. stray dog population and vector distribution) were shown to be associated with VL in previous studies, these factors were not used in the present study due to the availability of data. In addition, a sample set of places where VL was not observed during 2005–2015 was used to generate pseudo-absence data due to the difficulties of estimating real absence records. In the future investigations, we will increase the collection of relevant data and generate pseudo-absence data based on some other metric.

The multi-year mean values of related factors reflecting relatively stable environmental and socioeconomic conditions were adopted as input features for the ensemble BRT models. Therefore, the final predicted map represents the long-term average risk of VL infection in the Xinjiang Uygur Autonomous Region. The distribution of VL cases reported from 2016 to 2017 is shown in Additional file 1: Figure S3 shows. In 2016, 187 VL cases occurred in the predicted high-risk areas, and only 6 VL cases occurred in the predicted low-risk areas. In 2017, 42 VL cases occurred in the Xinjiang Uygur Autonomous Region, and only 1 VL case occurred in the predicted low-risk areas. It is important noted the global temperature is rising continuously with greenhouse gas emissions, and some changes may occur in related environmental and socioeconomic factors. In future research, we will combine a regional atmospheric circulation model with BRT modelling technology to recompute the potential endemic foci for the years 2030 and 2050 in the Xinjiang Uygur Autonomous Region under specific climate warming scenarios.

## Conclusions

Our findings show that land cover, urban accessibility, night-time light, mean temperature and NDVI are the important predictors contributing to the occurrence map. Approximately 16.64 million people inhabited the predicted potential infection risk zones in the Xinjiang Uygur Autonomous Region. The medical resources of the region are relatively scarce. This study provides a better understanding of the potential endemic foci of VL in the Xinjiang Uygur Autonomous Region with a 1 km spatial resolution, thereby enhancing our capacity to target the potential risk areas, to develop disease control strategies and to allocate medical supplies.

## Supplementary information

**Additional file 1: Table S1.** Land cover types. **Table S2.** Population living in areas with high predicted VL risk within each prefecture-level administrative unit and the top six regions contributing to these populations at risk. **Figure S1.** The predicted potential VL infection risk zones and the boundaries of the main administrative districts. **Figure S2.** Uncertainty in the model of predicted VL infection risk. **Figure S3.** The distribution of VL cases reported from 2016 (**a**) to 2017 (**b**).

## Data Availability

All relevant data are contained within the paper and its additional file.

## Abbreviations

VL: visceral leishmaniasis
BRT: boosted regression tree
REDCP: Resource and Environment Data Coud Platform
GDAL: geospatial data abstraction library
NDVI: normalized difference vegetation index
GIMMS: global inventory modelling and mapping studies
ESA: European Space Agency
CMDC: China Meteorological Data Service Center
SRTM: Shuttle Radar Topography Mission
ECJRC: European Commission Joint Research Center
NOAA: National Oceanic and Atmospheric Administration
AVHRR: advanced very high-resolution radiometer
CDC: Chinese Center for Disease Control and Prevention
AUC: area under curve
RC: relative contribution
SE: standard error
