# Supplementary material for: Risk factors and predicted distribution of visceral leishmaniasis in the Xinjiang Uygur Autonomous Region, China, 2005–2015
Source: Parasit Vectors. 2019 Nov 8;12:528. doi: 10.1186/s13071-019-3778-z (PMC6839266; doi:10.1186/s13071-019-3778-z)
Supplement: Supplementary file 1 — Additional file 1: Table S1. Land cover types. Table S2. Population living in areas with high predicted VL risk within each prefecture-level administrative unit and the top six regions contributing to these populations at risk. Figure S1. The predicted potential VL infection risk zones and the boundaries of the main administrative districts. Figure S2. Uncertainty in the model of predicted VL infection risk. Figure S3. The distribution of VL cases reported from 2016 (a) to 2017 (b). [file 13071_2019_3778_MOESM1_ESM.docx]

**Additional file 1: Table S1.** The names of the land cover types.

| **Value** | **Label** |
| --- | --- |
| 1 | Post-flooding or irrigated croplands (or aquatic) |
| 2 | Rainfed croplands |
| 3 | Mosaic cropland (50-70%) / vegetation (grassland/shrubland/forest) (20-50%) |
| 4 | Mosaic vegetation (grassland/shrubland/forest) (50-70%) / cropland (20-50%) |
| 5 | Closed (>40%) broadleaved deciduous forest (>5m) |
| 6 | Closed (>40%) needleleaved evergreen forest (>5m) |
| 7 | Open (15-40%) needleleaved deciduous or evergreen forest (>5m) |
| 8 | Closed to open (>15%) mixed broadleaved and needleleaved forest (>5m) |
| 9 | Mosaic forest or shrubland (50-70%) / grassland (20-50%) |
| 10 | Mosaic grassland (50-70%) / forest or shrubland (20-50%) |
| 11 | Closed to open (>15%) (broadleaved or needleleaved, evergreen or deciduous) shrubland (<5m) |
| 12 | Closed to open (>15%) herbaceous vegetation (grassland, savannas or lichens/mosses) |
| 13 | Sparse (<15%) vegetation |
| 14 | Closed to open (>15%) grassland or woody vegetation on regularly flooded or waterlogged soil - Fresh, brackish or saline water |
| 15 | Artificial surfaces and associated areas (Urban areas >50%) |
| 16 | Bare areas |
| 17 | Water bodies |
| 18 | Permanent snow and ice |

**Additional file 1: Table S2.** Population living in areas with high predicted VL risk within each prefecture-level administrative unit and the top six regions contributing to these populations at risk.

| **Region** | **Population living in high-risk zones (millions)** |
| --- | --- |
| Kashgar Prefecture | 3.95 |
| Urumqi City | 3.69 |
| Ili Kazakh Autonomous Prefecture | 1.94 |
| Aksu Prefecture | 1.73 |
| Hotan Prefecture | 1.19 |
| Bayingolin Mongol Autonomous Prefecture | 1.00 |
| **Xinjiang Uygur Autonomous Region** | **16.64** |

**
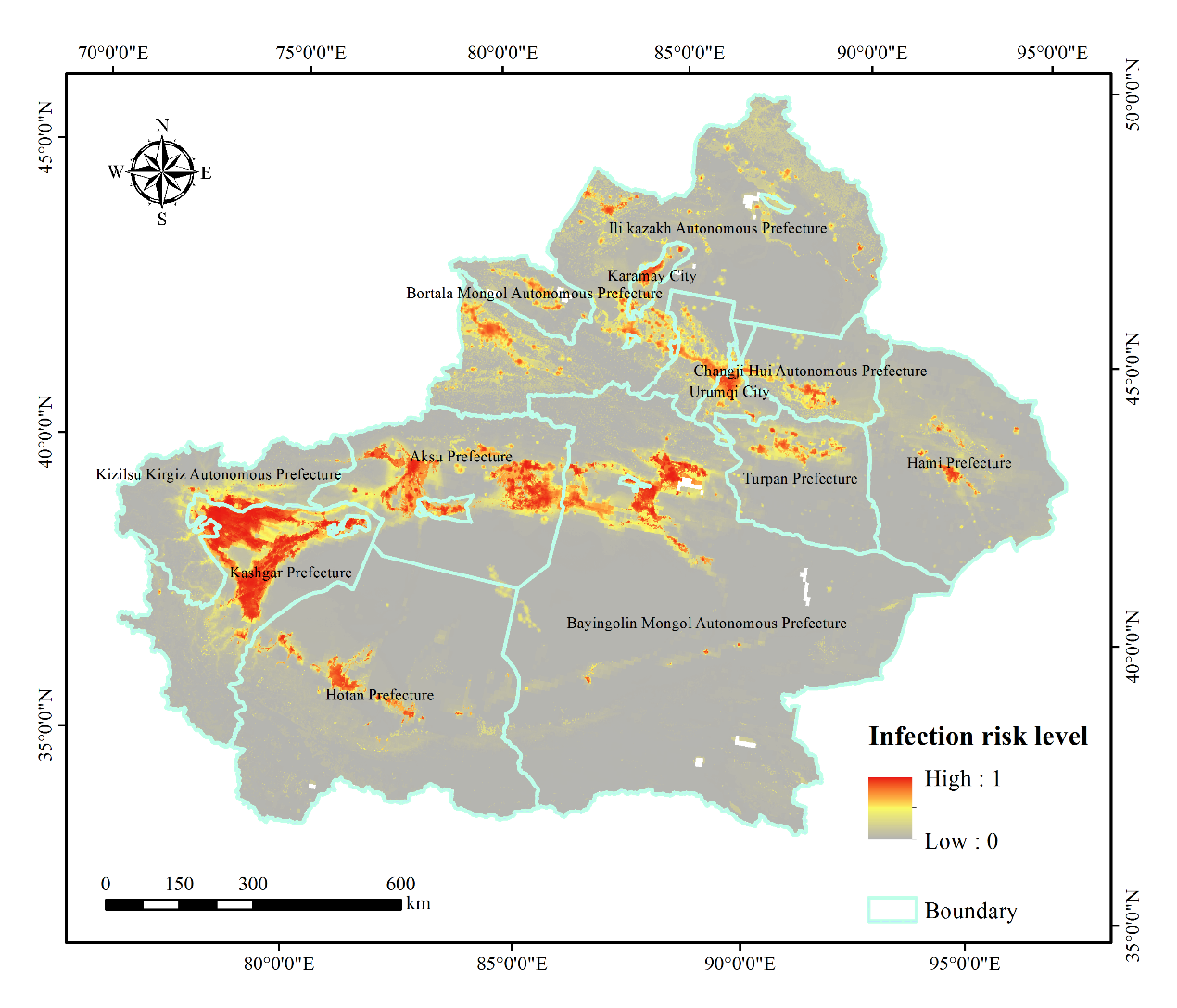
**

**Additional file 1: Figure S1.** The predicted potential VL infection risk zones and the boundaries of the main administrative districts.

**
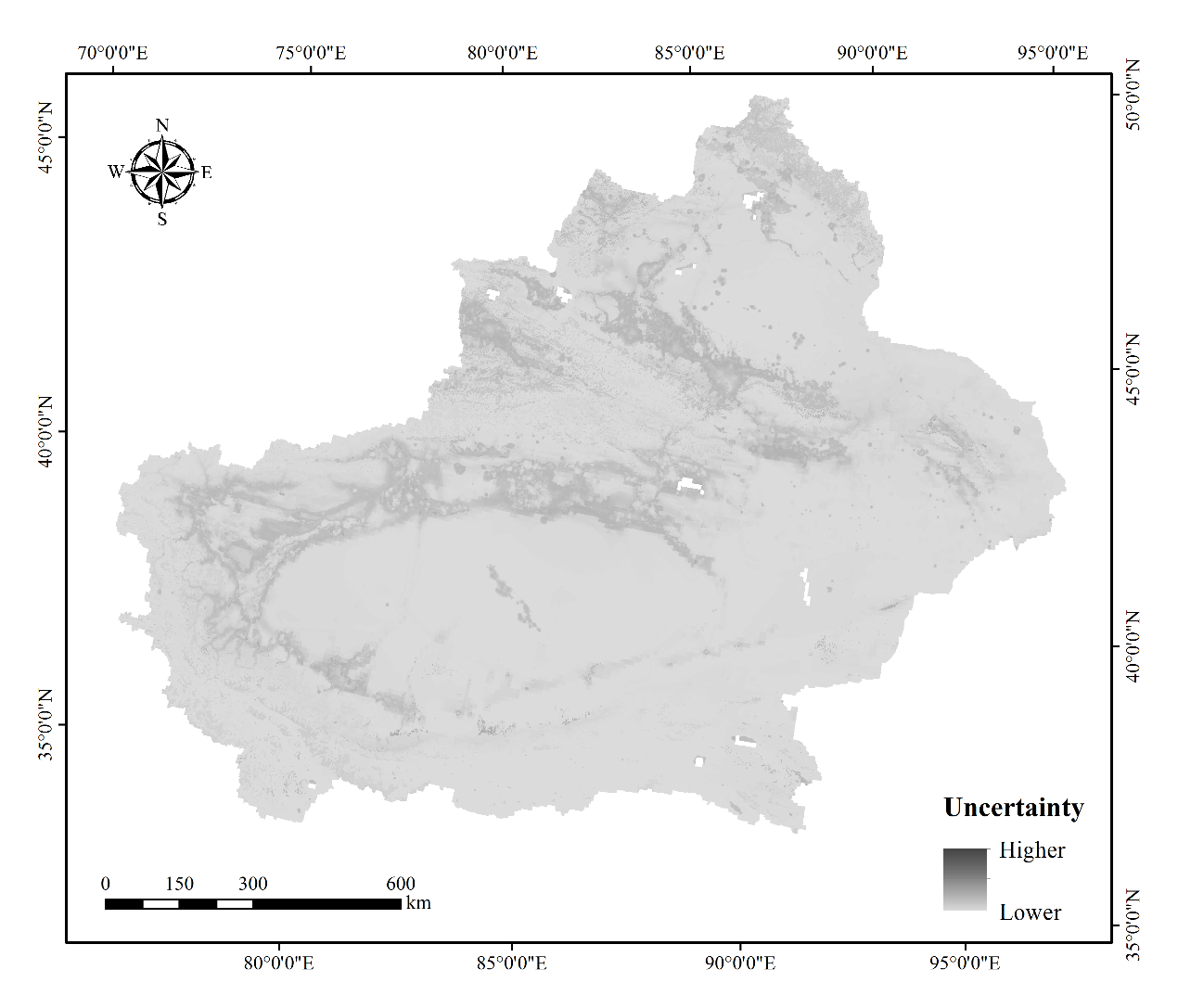
**

**Additional file 1: Figure S2.** Uncertainty in the model of predicted VL infection risk.


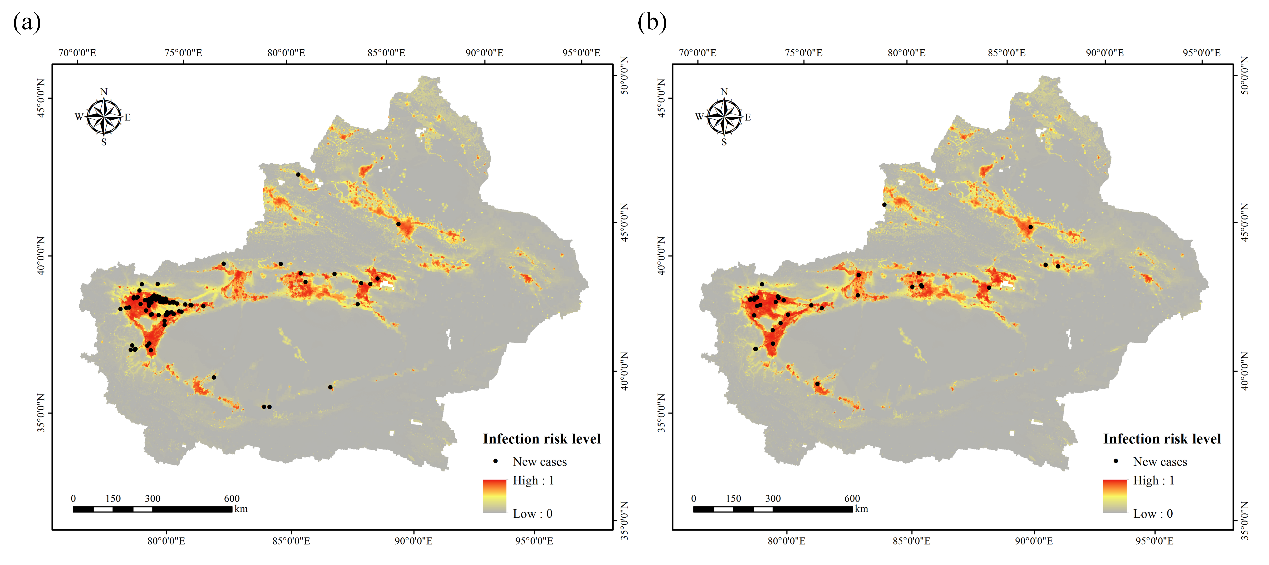


**Additional file 1: Figure S3.** The distribution of VL cases reported from (a) 2016 to (b) 2017.
